# Effects of oral meloxicam on physiological and behavioral outcomes of weaned calves following band castration

**DOI:** 10.1093/tas/txaf094

**Published:** 2025-07-22

**Authors:** J D Garcia, B K Whitlock, P D Krawczel, J A Carroll, N C Burdick Sanchez, J W Dailey, J A Daniel, J F Coetzee

**Affiliations:** Large Animal Clinical Sciences, College of Veterinary Medicine, The University of Tennessee, Knoxville, TN, 37996, USA; Large Animal Clinical Sciences, College of Veterinary Medicine, The University of Tennessee, Knoxville, TN, 37996, USA; Department of Animal Science, The University of Tennessee, Knoxville, TN, 37996, USA; Department of Animal Science, Faculty of Agricultural Sciences, University of Helsinki, Helsinki, 00014, Finland; Production Animal Medicine, Faculty of Veterinary Medicine, University of Helsinki, Helsinki, 00014, Finland; United States Department of Agriculture-Agricultural Research Service, Livestock Issues Research Unit, Lubbock, TX, 79403, USA; United States Department of Agriculture-Agricultural Research Service, Livestock Issues Research Unit, Lubbock, TX, 79403, USA; United States Department of Agriculture-Agricultural Research Service, Livestock Issues Research Unit, Lubbock, TX, 79403, USA; Department of Animal Science, Berry College, Mt. Berry, GA, 30149, USA; Department of Anatomy and Physiology, Kansas State University, Manhattan, KS, 66506, USA

**Keywords:** analgesia, band castration, meloxicam, pain behavior, post-weaned calves, welfare

## Abstract

Castration detrimentally affects weaned calves, and painful procedures in production animals are a public concern. The objective of this study was to determine the effects of castration (by banding) with or without administration of meloxicam (Mel), a non-steroidal anti-inflammatory drug in weaned beef calves. Forty-eight (62 d post-weaning) beef calves [8.2 ± 0.1 (mean ± SE) mo old; 319 ± 10 kg BW] were blocked by age and body weight and randomly assigned to 1 of 3 treatments (n = 16 calves per treatment): 1) intact bulls (BULL), 2) castration by banding (BAN), or 3) castration by banding with orally-administered Mel (3 mg per kg BW on d 0 and 14; BAN + M). Within each treatment group, calves were randomly assigned to 8 pens (2 calves per treatment within each pen). Body weight and plasma haptoglobin and fibrinogen concentrations were determined on 0, 3, 7, 14, and 28 d after treatment administration. Rectal temperature was recorded at 5-min intervals for the first 14 d by dataloggers. Behaviors [mean lying time (h/d), mean lying bouts (n/d), and steps (n/d)] were recorded at 1-min intervals for 27 d by dataloggers. Ethogram data was recorded on 8 d for two hours with collection times of every 10 min. Behaviors recorded from the ethogram included eating, ruminating, not ruminating, drinking, location within the pen, and body position (standing or lying down). Data were tested for effects of treatment, day, pen, and treatment by day interaction using mixed models accounting for repeated measures. BULL gained more (0.69 ± 0.12 kg/d; P < 0.05) than BAN (0.15 ± 0.11 kg/d) or BAN + M (0.14 ± 0.11 kg/d) over 28 d. There was an effect of treatment (P < 0.001) and treatment by time interaction (P < 0.001) on mean rectal temperature during the 14 d after treatment administration. Over 14 d, BAN + M had the greatest mean rectal temperature (39.47 ± 0.006 °C), BAN had the second greatest temperature (39.42 ± 0.006 °C), and BULL had the lowest temperature (39.41 ± 0.005 °C). BULL increased time lying (P < 0.05) and decrease steps (P < 0.05), compared to BAN, Days 2, 3, 16, and 17, and compared to BAN and BAN + M Days 18 and 19 post-castration. Mel administration had an insignificant effect on pen-level behaviors recorded with the ethogram. Decreased weight gain indicates that castration by banding during the post-weaning period was painful regardless of attempts and pain abatement with Mel. While benefits of Mel were not evident from changes in growth or inflammatory response, behavior and rectal temperature were affected by Mel administration.

## Teaser

This study highlights that oral meloxicam administered to post-weaned calves prior to band castration can reduce pain-associated behaviors. However, meloxicam did not significantly impact growth or systemic inflammatory markers, suggesting further research is needed to refine dosing for optimal pain relief and potential production benefits.

## INTRODUCTION

Castration is commonly completed on cattle in the U.S. without analgesia (Coetzee et al., 2010; Coetzee, 2011). Painful procedures are a rising public concern because of greater awareness of animal health and well-being (Broom, 2010; Alonso et al., 2020). Currently, there is only one compound [Banamine® Transdermal (flunixin transdermal solution)] specifically approved for analgesic use, control of pain associated with infectious pododermatitis, also known as foot rot, in cattle in the U.S. (Kleinhenz et al., 2016). In Canada, approved label indications for meloxicam (Mel) for use in pain management include injectable and oral suspension for dehorning and castration (both band and surgical), respectively, sparking interest in its potential for pain mitigation during castration in the U.S. (Gonzalez et al., 2010; Coetzee, 2011; Olson et al., 2016). Extra label non-steroidal anti-inflammatory drug (NSAID; e.g., Mel) use has positive effects on certain stress physiology and the productivity of calves following surgical and band castration (Webster et al., 2013; Brown et al., 2015; Olson et al., 2016).

Oral Mel suspension administered two hours prior to band and surgical castration in 4 to 5-mo-old Holstein bulls decreased plasma cortisol and substance P as well as heart rates and also had a significant effect on behavioral scores including increased number of steps and decreased percent of time lying and lying bouts when compared to calves without Mel (Olson et al., 2016). Improved average daily gain and reduced pen-level first pull rate (removing animals from their pen for health-related issues) was demonstrated following oral administration of Mel 24 hours prior to surgical castration in weaned feedlot calves (Coetzee et al., 2012). In addition, Mel is a cyclooxygenase (COX) 2 selective NSAID and thus embody a favorable COX2:1 ratio, which is advantageous in preventing adverse events (Griswold and Adams, 1996).

To improve animal welfare and respond to increasing calls for the routine inclusion of analgesics in castration protocols, there is a critical need for regulatory approval of a safe, cost effective, and practical pain-alleviating compound (Coetzee, 2013; Canozzi et al., 2017). An ideal candidate would not only provide effective pain relief but also support or enhance production parameters. Despite promising research in calves, few studies have been done that examine the effects of Mel use during bloodless (banding) castration in specifically post-weaned calves. The hypothesis of this study was that oral administration of Mel would alleviate some of the negative physiological and behavioral effects associated with banding castration in post-weaned beef calves. The objectives were to evaluate the effects of oral Mel on body weight gain, markers of inflammation, rectal temperature, and behavioral indicators of pain and discomfort following castration.

## MATERIALS AND METHODS

### Animals, Housing, and Management

The study design and all animal handling procedures were approved by the Institutional Animal Care and Use Committee at the University of Tennessee in Knoxville, Tennessee (UTK-IACUC Number: 2070 v 12 2 11). Angus bull calves [n = 48; 8.2 ± 0.1 (mean ± SE) mo old; 322 ± 6 kg initial BW] from the research cow-calf herd at the East Tennessee AgResearch and Education Center (Louisville, TN, USA; Latitude = 35.843479; Longitude = −83.955234) were enrolled in this trial during Fall time (October and November). The eligible population of calves was selected by the farm manager. Only calves that were not intended to be kept as bulls or sold for breeding purposes were included in the study. The only exclusion criteria were related to the general health of the calves and the absence of cryptorchidism. Initially, 52 calves were provided, from which 48 were selected for the study. One calf was excluded due to a musculoskeletal injury, another because of cryptorchidism, and one was excluded because its body weight was nearly 35 kg greater than that of the next heaviest calf. A third calf was randomly excluded to reach the target number of 48.

Prior to study enrollment, all calves were tested for persistent infection with bovine viral diarrhea virus using antigen capture enzyme-linked immunosorbent assay on ear notch samples. All animals tested negative for bovine viral diarrhea virus. In accordance with standard farm protocols, all calves received initial and booster vaccinations and anthelmintic treatment approximately 2 mo prior to study initiation, around the time of weaning. Vaccinations included a 10-way respiratory and reproductive vaccine targeting common viral pathogens and *Leptospira* spp. (*Bovi-Shield GOLD FP® 5 L5*, Zoetis Inc., Kalamazoo, MI, USA), a 7-way clostridial vaccine (*Vision® 7 with SPUR®*, Merck Animal Health, Madison, NJ, USA), and moxidectin as an anthelmintic (*Cydectin® Pour-On*, Boehringer Ingelheim Vetmedica Inc., St. Joseph, MO, USA). To reduce the risk of tetanus associated with banding castration, all calves also received initial and booster doses of a clostridial vaccine containing *Clostridium tetani* toxoid (*Bar-Vac® CD/T*, Boehringer Ingelheim Vetmedica Inc., St. Joseph, MO, USA), administered approximately 14 d prior to and on the day of treatment.

Once enrolled, calves were excluded from the study if they exhibited any of the following conditions at any point during the experimental period: (1) a body weight loss exceeding 10% relative to baseline (Day 0), (2) clinical illness, whether directly or indirectly associated with the experimental treatment, that progressed to a life-threatening condition, (3) persistent anorexia, defined as failure to approach or consume feed from the bunk on a daily basis, (4) abnormal behavioral response characterized by failure to display typical inquisitive behavior, or (5) loss of mobility. Body weight was recorded on Days −7, 0, 3, 7, 14, and 28, while appetite, behavior, and mobility were assessed through direct observation daily throughout the study. Calves meeting any of these criteria would be removed from the study and provided appropriate veterinary care, however, no calves were removed from the study after inclusion.

### Experimental Design

Calves were weaned 62 d before experimental treatment administration. All calves were weighed (Digi-Star Stock Weigh 600 with 24” load cells mounted to an aluminum platform), and wither height and scrotal circumference determined (Reliabull Scrotal Tape®, Lane Manufacturing Inc.) prior to treatment administration (Day −7). Calves were first divided by source herd (two herds within the farm, with an equal number of calves from each included in the study). Within each herd, calves were ranked by age and then by body weight. Groups of three calves with similar age and body weight were then formed, and within each group, calves were randomly assigned to one of the three treatment groups [n = 16 calves per treatment: 1) intact bulls (sham handled/castration mimicked; BULL; 8.1 ± 0.1 mo old; 319 ± 10 kg initial BW), 2) castration by banding (The Callicrate Bander®, No Bull Enterprise, St. Francis, Kansas, USA; BAN; 8.2 ± 0.1 mo old; 323 ± 11 kg initial BW), or castration by banding with orally-administered Mel (15 mg tablets; ZyGenerics, Cadila Healthcare Ltd, NDC 68382-051-05; 3 mg/kg BW on Day 0 and 14; BAN + M; 8.3 ± 0.1 mo old; 324 ± 8 kg initial BW)]. This approach resulted in an approximately equal distribution of calves from each herd across treatment groups, with similar age and body weight profiles among the groups. Individual does of Mel ranged from 2.98 to 3.02 mg/kg (mean of 2.99 and 3.01 mg/kg on Day 0 and 14, respectively) as tablets were left whole to preserve the integrity of the drug.

Mel was administered by inserting tablets (by rounding to the nearest whole Mel tablet) into a porcine gelatin capsule (Torpac Veterinary Size Capsules, 10 mL capsule, Torpac by Custom Capsules Ltd.) prior to being administered to the animal *per os* using a stainless-steel balling gun. In this experiment the BULL and BAN groups received empty porcine gelatin capsules administered *per os* also using a balling gun. Calves were monitored for approximately 1 min to ensure they swallowed the boluses.

**Figure 1.**
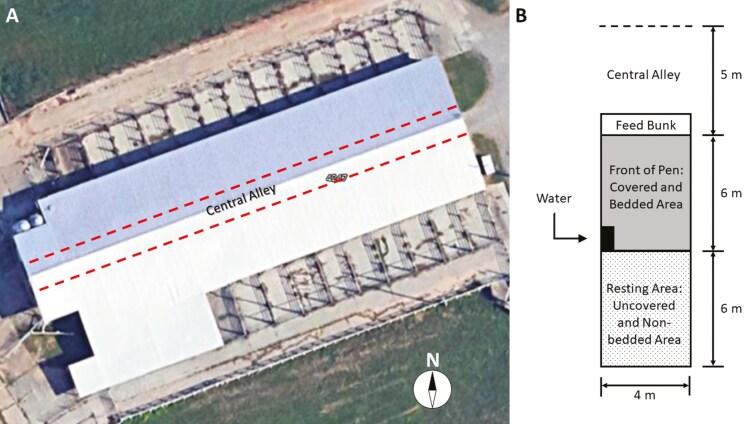
Study site and pen layout. (A) Calves were housed at the East Tennessee AgResearch and Education Center–Blount Unit and assigned to 8 pens (6 calves/pen; 2 calves/treatment: Bull, BAN, BAN + M). Four pens were located on each side of a central alley (dashed lines). Calves acclimated from Day −7 and remained in the pens through Day 28, except during treatments, sampling, or weighing. The compass rose indicates north. (B) Pen schematic: Each 4 × 12 m pen had a front feed bunk with steel cables, a 6 m covered, bedded area with a shared automatic waterer at the back, and a 6 m uncovered, concrete-floored area with access to an exit alley.

Calves were assigned to 8 pens (Figure 1; 2 calves per treatment within each pen) one wk before treatment administration for acclimation to group housing and maintained in these pens for the duration of the experiment (Day −7 to 28) (Google Maps, 2024). Within their respective pens, calves were offered approximately 4.8 kg (DM) of corn silage and approximately 1.0 kg (DM) grass hay once daily (0800 hour) per calf and allowed ad libitum access to water.

### Growth Rate Assessment

Body weight was measured on Day −7, 0, 3, 7, 14, and 28 as described above to determine average daily body weight gain (ADG). To calculate ADG, the total weight gained, in a period, was divided by the number of days between weigh-ins.

### Plasma Meloxicam, Fibrinogen, and Haptoglobin Concentrations

On Day 0, 3, 7, 14, and 28, 10 mL blood samples were collected (in K2EDTA reference 367841 and Lithium Heparin reference 367880; BD Vacutainer Blood Collection Tubes) from the jugular vein of each calf prior to the administration of any treatment. Blood samples were placed immediately on ice until plasma separation using centrifugation (3,000 X *g*; 10-min) was completed less than 6 hours following blood collection. Plasma was harvested from the blood and stored at −80 °C until analysis.

All plasma samples were analyzed for Mel, haptoglobin, and fibrinogen concentrations. Plasma concentrations of Mel were determined by high pressure liquid chromatography (Surveyor MS Pump and Autosampler; Thermo Scientific, San Jose, CA) with mass spectrometry detection (TSQ Quantum Discovery MAX; Thermo Scientific; HPLC-MS) at Iowa State University in Ames, Iowa (Kreuder et al., 2012; Repenning et al., 2013). The standard curve in bovine plasma was linear from 0.005 to 10.0 ug/mL. The correlation of coefficient exceeded 0.995 and all measured values were within 15% of the actual values with most less than 5% difference from the actual values. The accuracy of the assay for Mel in bovine plasma was 99 ± 3% of the actual concentration whereas the coefficient of variation was 5% determined on 4 sets of replicates for each of the following concentrations: 0.015, 0.15, and 1.5 μg/mL. The limit of quantitation (LOQ) for this assay was 0.005 ug/mL, while the limit of detection (LOD) was 10-fold lower than that at 0.0005 ug/mL.

Plasma samples were analyzed for haptoglobin concentrations by the Kansas State University Veterinary Diagnostic Laboratory in Manhattan, Kansas. Plasma haptoglobin concentrations were determined using a Roche Cobas c501 biochemistry analyzer (Roche Diagnostics, Laval, QC, Canada) using a Tridelta bovine haptoglobin calibrator (TP801CAL, Tridelta, Maynooth, Ireland) and 2 levels of in-house controls (bovine serum pools; low = 59 mg/dL; high = 130 mg/dL). The intra-and inter-assay coefficient of variations were 5.3% to 6.3% and 5.7% and 4.1% for the low and high controls, respectively, with the analytical sensitivity was determined as 0.5 mg/dL (Marti et al., 2017).

Fibrinogen concentrations were measured using the heat precipitation method, at the University of Tennessee College of Veterinary Medicine in Knoxville, Tennessee, which remains a widely used and practical technique in bovine medicine for detecting inflammatory responses. While semi-quantitative in nature, this method has an estimated LOD of approximately 100 to 150 mg/dL and a LOQ of 150 to 200 mg/dL. Concentrations below this range are difficult to detect or quantify reliably, whereas values above ~200 mg/dL fall within the method’s reportable range and can be interpreted with greater confidence (Millar et al., 1971; Kaneko et al., 2008; Weiss and Wardrop, 2011; Thrall et al., 2012).

### Behavioral Assessment

On Day 0, IceTags™ (IceRobotics, Inc. Edinburgh, Scotland) were placed on each animal. The IceTag™ is a 3-axis accelerometer datalogger that records behavior (standing, lying, stepping, and motion index of the animal) at 1-min intervals that may be stored for up to 60 d. IceTags™ were affixed to the right hind limb by loosely fitting the band around the distal metatarsal region of the limb on Day 0 and removed on Day 28.

An ethogram was developed for this study by which some activities and behaviors of the calves could be determined and subsequently analyzed. Throughout the experiment, the behavioral observations were conducted by four individuals affiliated with the study. All observers contributed to the development of the ethogram prior to the observation period. During this process, the team met to define each behavioral category and discussed specific examples to ensure consistent interpretation and minimize subjective bias. These discussions served as the primary method of calibration. Though no formal inter-observer reliability test was performed, this collaborative development and consensus-building process aimed to enhance consistency in data collection. Additionally, all observers completed at least one ethogram, two hours of observations of 4 pens with 6 calves in each pen, within a week of the onset of the study.

On Day 2, 3, 4, 5, 7, 8, 15, and 29 calves were observed in their pens by one individual (visible to the cattle) from 0800 to 0950 hour and the ethogram was completed for each calf every 10 min. The individual completing the ethogram was blinded to Mel treatment but not BULL or BAN, as these treatments would be easily noticeable. While the animals were acclimated to people in Central Alley, where observations were made from and near the Feed Bunk, during the acclimation period, animals were not acclimated to the particular observer. Ethograms assessed three aspects of calf activity and behavior; 1) activities associated with their mouth (eating, ruminating, not ruminating, or other), 2) location within the pen (Figure 1) resting area, front of pen, or bunk, and 3) position (standing or lying down) (Google Maps, 2024). Ethogram data were summarized by the hour of observation into the 0800 hour and the 0900 hour for statistical analysis.

### Rectal and Ambient Temperature Assessment

**Figure 2.**
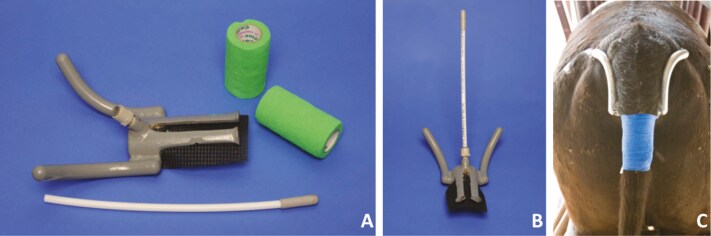
Device to monitor rectal temperature automatically in cattle. A) The tail harness and probe container are custom-machined aluminum, the probe was a micro-T temperature logger (Star Oddi, Iceland). The quick connect and PEX tubing are commercially available plumbing parts. B) Assembled device, prior to installation, used to automatically monitor rectal temperature in cattle. C) Device installed on a beef steer monitoring rectal temperature.

Rectal temperature was recorded at 5-min intervals for 14 d by dataloggers fitted to the calves on Day 0 (Reuter et al., 2010; Figure 2). The tail harness and probe container were custom-machined aluminum, the probe was a micro-T temperature logger (Star Oddi, Iceland). One HOBO pendant temperature logger (HOBO Data Loggers, Bourne, MA, USA) was placed in the barn in which the calves were housed (approximately in the middle of the pens; approximately 1 meter above the heads of standing calves) on Day 0 and removed 14 d later for measurement of environmental temperature.

### Sample-Size Consideration and Data Collection

Sample size determination was informed by previous literature, anticipated treatment effects, and estimated standard deviations, in conjunction with the number of animals available for the study (approximately 50 in total). These factors guided the allocation of animals into the most logical and balanced treatment groups. While greater statistical power is always preferable and our findings should be interpreted with appropriate consideration of these limitations, we did observe notable differences among treatment groups. Although some comparable studies have employed larger group sizes and achieved higher power, others have utilized similar or even fewer animals per group and still produced informative and impactful results (Repenning et al., 2013; Roberts et al., 2015; Daniel et al., 2020; Gellatly et al., 2021).

Data collection was conducted on-farm using pen-and-paper methods for recording individual animal information, including body weight, age, behavioral observations (ethogram data), and other in vivo parameters. Upon completion of each data collection period, paper records were transported to the laboratory and transcribed into structured Microsoft Excel spreadsheets following a consistent and logical organization. Data entry was performed on a secure laboratory computer and backed up to the University of Tennessee’s Microsoft OneDrive cloud storage system in accordance with institutional data management protocols. Laboratory-generated data, including circulating concentrations of haptoglobin, fibrinogen, and Mel, rectal temperatures, and activity data collected via IceTags™, were directly entered into Excel spreadsheets and similarly backed up to cloud storage. All datasets were subsequently imported into statistical software for analysis.

### Statistics

All statistical analyses were conducted using JMP (version 15, SAS Institute Inc., Cary, NC) and SAS (version 9.4, SAS Institute Inc.). The analytical unit for all outcomes was the individual calf unless otherwise noted.

ADG was calculated for four discrete periods: Day 0 to 7, Day 7 to 14, Day 14 to 28, and cumulatively for Day 0 to 28. Each period was treated as a separate outcome and analyzed using a repeated measures linear mixed model (MIXED procedure in JMP). Treatment and period were included as fixed effects, and calf nested within treatment was included as a random effect. The interaction between treatment and period was also evaluated. Pairwise comparisons among treatment groups (BULL, BAN, BAN + M) were completed using Tukey’s Honestly Significant Difference (HSD) test when main effects or interactions were significant (P < 0.05). Residuals were assessed for normality using the Shapiro–Wilk test and Q–Q plots; homogeneity of variance was evaluated using residual vs. predicted plots. Assumptions were reasonably met.

Plasma concentrations of Mel, fibrinogen, and haptoglobin were analyzed as continuous outcomes using repeated measures linear mixed models. Treatment, day, and their interaction were included as fixed effects; calf nested within treatment was treated as a random effect. Post-hoc pairwise comparisons were conducted using Tukey’s HSD when appropriate. Model assumptions were verified as described above.

Behavior data were collected via IceTags™ worn on the right rear leg from Day 0 to Day 28. Due to variable timing of IceTag™ application and removal, behavior data from Days 0 and 28 were excluded. Raw 1-min data were aggregated to daily summaries for each calf. Lying time (h/d) and steps per day were treated as continuous variables and analyzed using repeated measures linear mixed models, with treatment, day, and their interaction as fixed effects, and calf nested within treatment as a random effect. Lying bouts per day, though a count variable, were initially assessed for overdispersion and fit using Poisson and negative binomial distributions. However, as residuals met assumptions for normality and homoscedasticity, a standard linear mixed model was used for consistency in behavioral endpoint analysis. In all models, significance was defined as P < 0.05. When significant effects were found, pairwise comparisons between all treatment groups were performed using Tukey’s HSD.

Rectal and ambient temperatures, recorded at 5-min intervals, were summarized by hour and analyzed using mixed model analysis of variance (PROC GLIMMIX, SAS). Ethogram data (standing, lying, walking, feeding behavior, location in pen, etc.) were similarly analyzed using PROC GLIMMIX. Fixed effects included treatment, day, and their interaction; random effects included pen and calf nested within pen. When appropriate, post-hoc comparisons were made using Fisher’s Least Significant Difference test (P < 0.05).

Rectal temperatures were also collapsed into daily binary outcomes representing febrile status (≥ 39.5 °C). A mixed effects logistic regression (PROC GLIMMIX with binary distribution and logit link) was used to assess the probability of being febrile. Fixed effects in this model included treatment, standing time, lying time, and environmental temperature. Calf was included as a random effect to account for repeated measures. Although this was a randomized controlled trial, inclusion of these variables allowed us to explore physiological drivers of febrile responses, which may inform future management strategies. Model diagnostics were performed for each outcome to ensure assumptions of normality, homoscedasticity, and independence were met. No transformations were required.

## RESULTS

### Growth Rate

**Figure 3.**
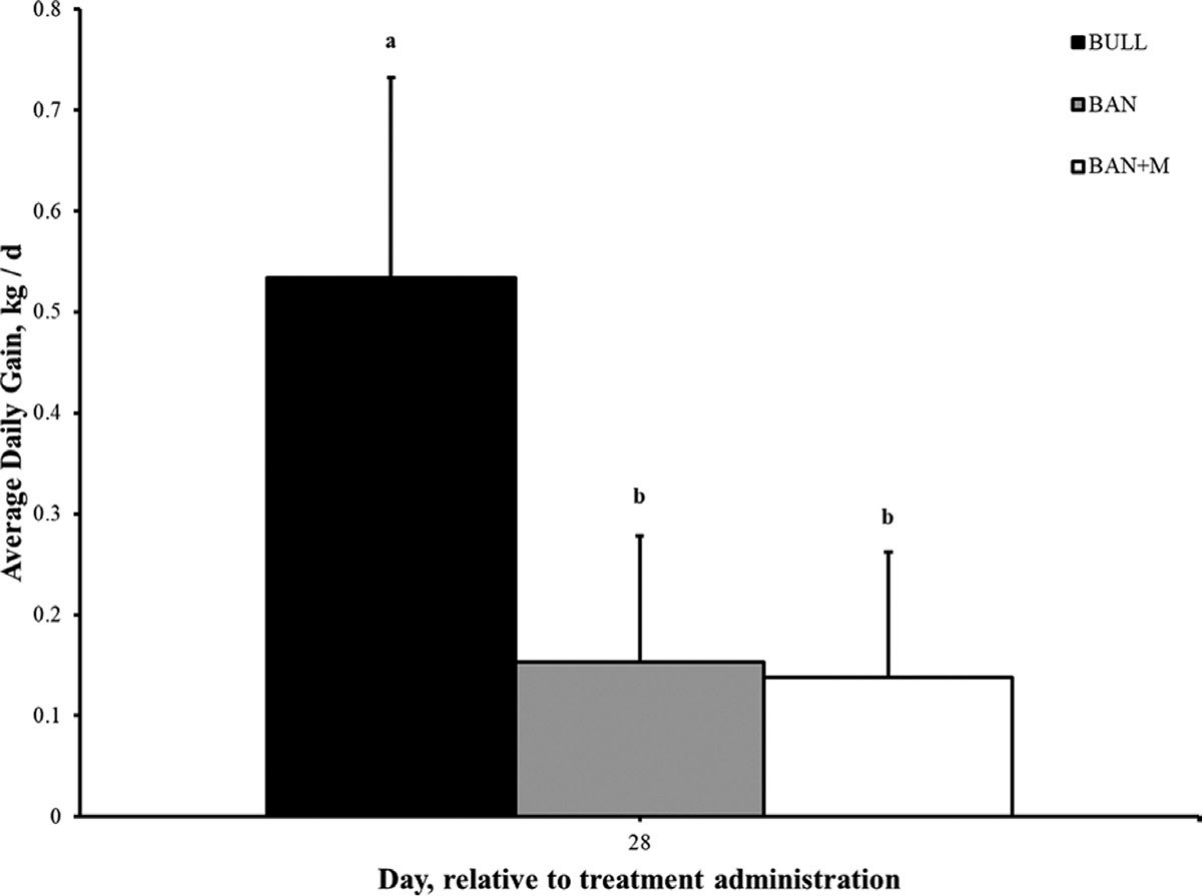
Average daily gain (kg/d + SEM) of weaned bulls (BULL), bulls castrated on Day 0 using a Callicrate Bander (BAN) and bulls castrated on Day 0 using a Callicrate Bander and administered meloxicam orally (3 mg/kg); BAN + M) on Day 0 and Day 14. There was an effect of treatment on average daily gain (P = 0.002) over 28 d. Values which do not share a common letter significantly differ (P < 0.05).

There was an effect of treatment (P = 0.002) on the overall ADG, from Day 0 to 28. BW gained in the BULL group was more [0.69 ± 0.12 (SEM) kg/d; P < 0.05] than BAN (0.15 ± 0.11 kg/d) or BAN + M (0.14 ± 0.11 kg/d) over 28 d (Figure 3). However, there was no effect of treatment (P ≥ 0.34) or interaction of treatment and period (P ≥ 0.91) on ADG for any other periods.

### Plasma Meloxicam, Fibrinogen, and Haptoglobin Concentrations

**Figure 4.**
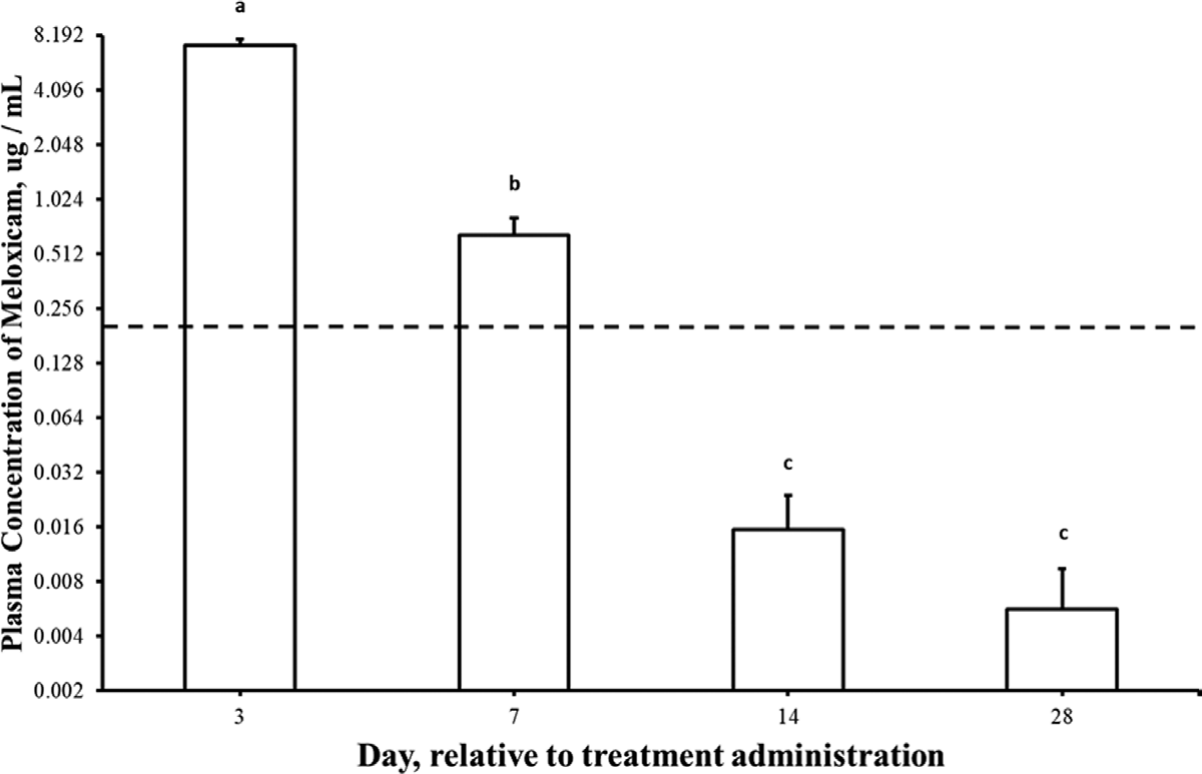
Plasma meloxicam concentrations (ug/mL + SEM) in weaned bulls castrated on Day 0 using a Calicrate Bander and administered meloxicam orally (3 mg/kg BW) on Day 0 and 14. Note that the y-axis is logarithmic scale base 2. The dashed line (0.2 ug/mL) is considered to be the minimum plasma Mel concentrations required for analgesic effects in other species. Values which do not share a common letter significantly differ (P < 0.05).

**Figure 5.**
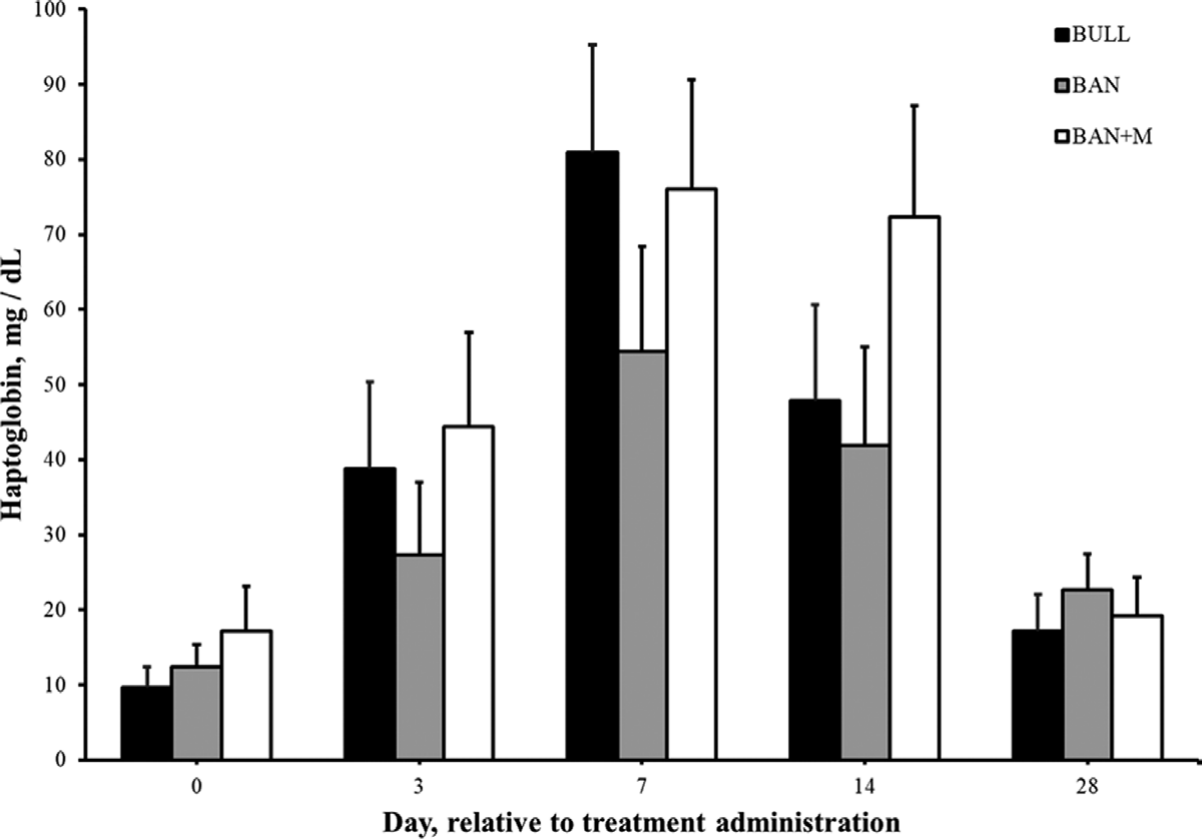
Plasma concentrations of haptoglobin (mg/dL + SEM) in weaned bulls (BULL), bulls castrated on Day 0 using a Callicrate Bander (BAN) and bulls castrated using a Callicrate Bander and administered meloxicam orally (3 mg/kg; BAN + M) on Day 0 and 14.

**Figure 6.**
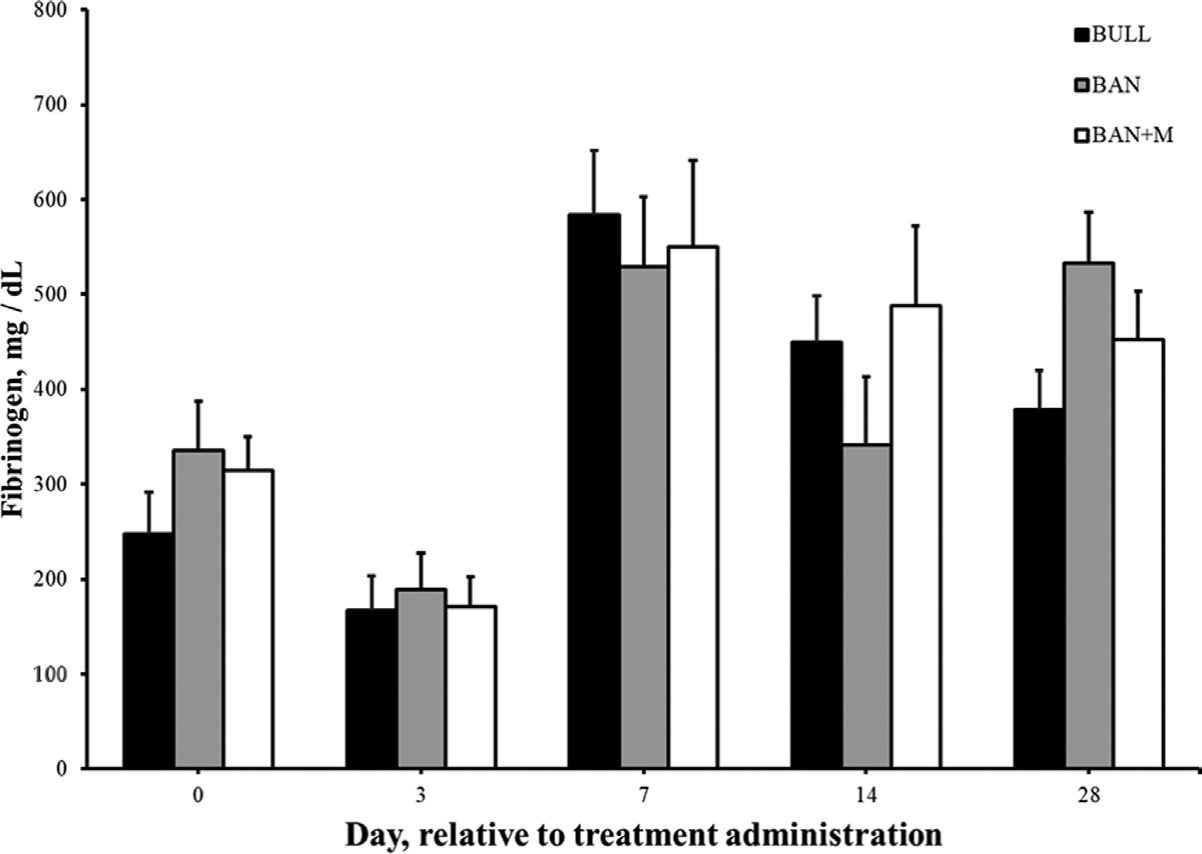
Plasma concentrations of fibrinogen (mg/dL + SEM) in weaned bulls (BULL), bulls castrated on Day 0 using a Callicrate Bander (BAN) and bulls castrated on Day 0 using a Callicrate Bander and administered meloxicam orally (3 mg/kg; BAN + M) on Day 0 and 14.

There was an effect of treatment (P < 0.0001) and an interaction of treatment and time (P < 0.0001) on plasma concentrations of Mel. No Mel was detected in blood samples from either BULL or BAN on Day 3, 7, 14, or 28. BAN + M plasma concentrations of Mel (Figure 4) was greatest on Day 3 (7.22 ± 0.57 µg/mL) and was still detectable on Day 7 (0.65 ± 0.15 µg/mL). While there was an effect of day (P < 0.0001), there was no effect of treatment (P = 0.36) or an interaction of treatment and day (P = 0.21) on mean plasma haptoglobin concentrations (Figure 5). Plasma concentrations of haptoglobin was greater (P < 0.05) on Day 7 (96.5 ± 27.5 mg/dL) than Days 0 (13.1 ± 2.4 mg/dL), 3 (36.9 ± 6.4 mg/dL), and 28 (19.7 ± 2.8 mg/dL) but not different (P > 0.05) than Day 14 (54.0 ± 7.9 mg/dL). Similarly, while there was an effect of day (P < 0.0001), there was no effect of treatment (P = 0.84) or an interaction of treatment and day (P = 0.25) on plasma fibrinogen concentrations (Figure 6). Plasma concentrations of fibrinogen was greater (P < 0.05) on Day 7 (554 ± 44 mg/dL) than Days 14 (426 ± 40 mg/dL), 0 (299 ± 25 mg/dL) and 3 (176 ± 20 mg/dL). Plasma fibrinogen concentrations was the least (P < 0.05) on Day 3.

### Behavior and Activity

**Figure 7.**
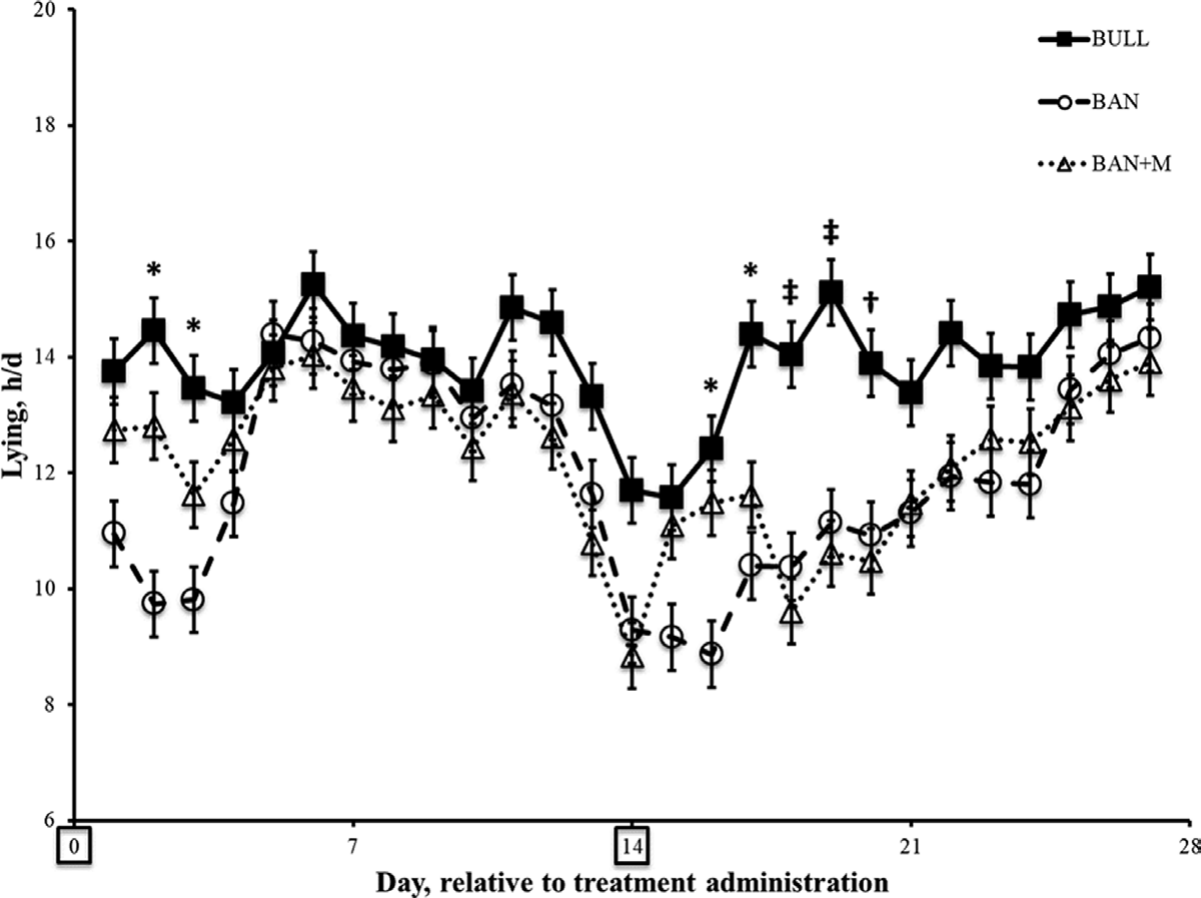
Lying time (h/d ± SEM) recorded by IceTags (a three-axis accelerometer) on weaned bulls (BULL; n = 16), bulls castrated on Day 0 using a Callicrate Bander (BAN; n = 16) and bulls castrated on Day 0 using a Callicrate Bander and administered meloxicam orally (3 mg/kg; BAN + M; n = 16) on Day 0 and 14 (boxed on the x-axis indicating the days of treatment). *Indicates BULL is greater than BAN on the same day (P < 0.05). ‡Indicates BULL is greater than BAN and BAN + M on the same day (P < 0.05). †Indicates BULL is greater than BAN + M on the same day (P < 0.05).

There was an effect of treatment (P = 0.0005), day (P < 0.0001) and an interaction of treatment and day (P < 0.0001) on lying time (h/d). Over the 27 d study period, BULL spent more time lying (13.9 ± 0.11 h/d) compared to BAN (11.9 ± 0.14 h/d; P < 0.05) or BAN + M (12.2 ± 0.11 h/d; P < 0.05), which did not differ from each other (P > 0.05). During the 3 and 6 d after the first (Day 0) and second (Day 14) administration of Mel, respectively, daily pairwise comparisons revealed differences among treatments. Specifically, on Days 2, 3, 16, and 17, BULL spent more time lying than BAN (P < 0.05) but not BAN + M (P > 0.05; Figure 7).

**Figure 8.**
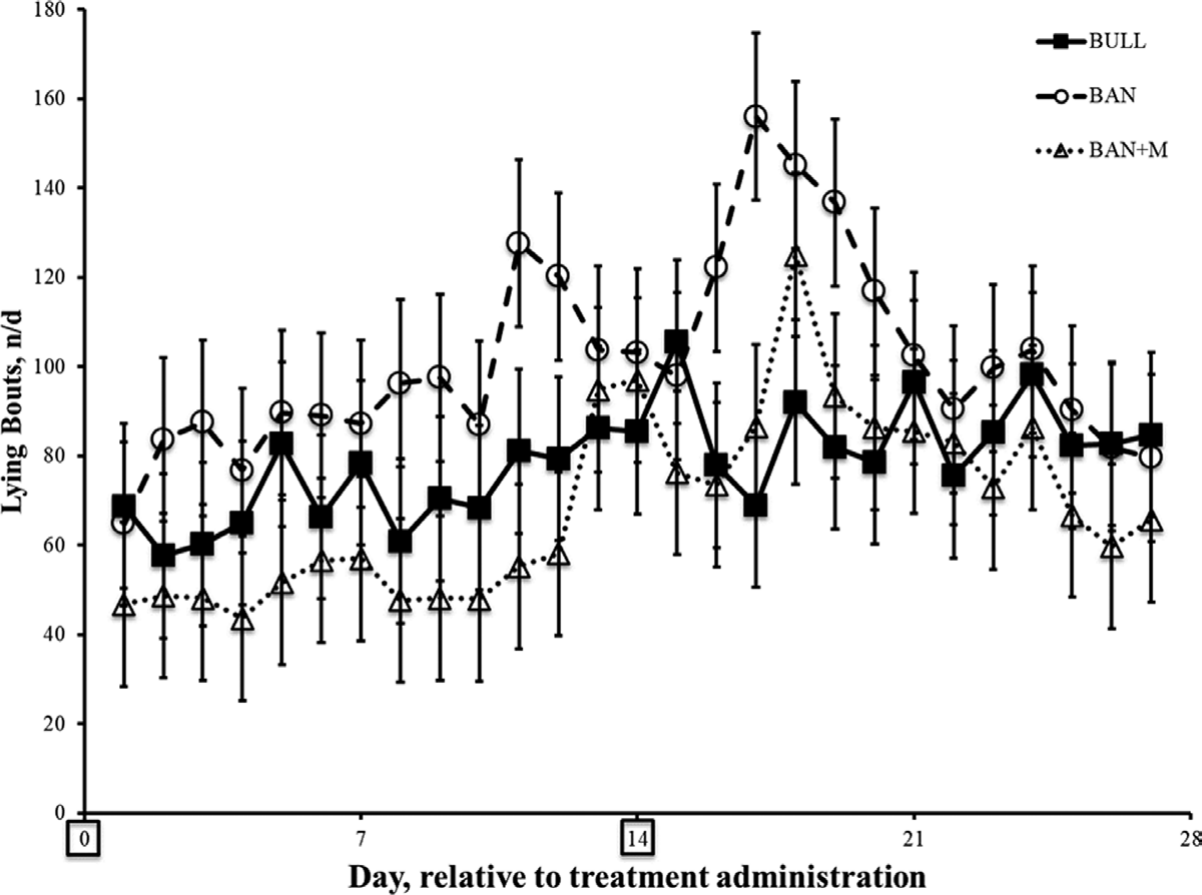
Lying bouts (n/d ± SEM) recorded by IceTags (a three-axis accelerometer) on weaned bulls (BULL; n = 16), bulls castrated on Day 0 using a Callicrate Bander (BAN; n = 16) and bulls castrated on Day 0 using a Callicrate Bander and administered meloxicam orally (3 mg/kg; BAN + M; n = 16) on Day 0 and 14 (boxed on the x-axis indicating the days of treatment).

For the number of lying bouts per day, there was an effect of day (P < 0.0001), but no effect of treatment (P = 0.23) or interaction of treatment and day (P = 0.38; Figure 8), so pairwise comparisons among treatments were not conducted for this variable.

**Figure 9.**
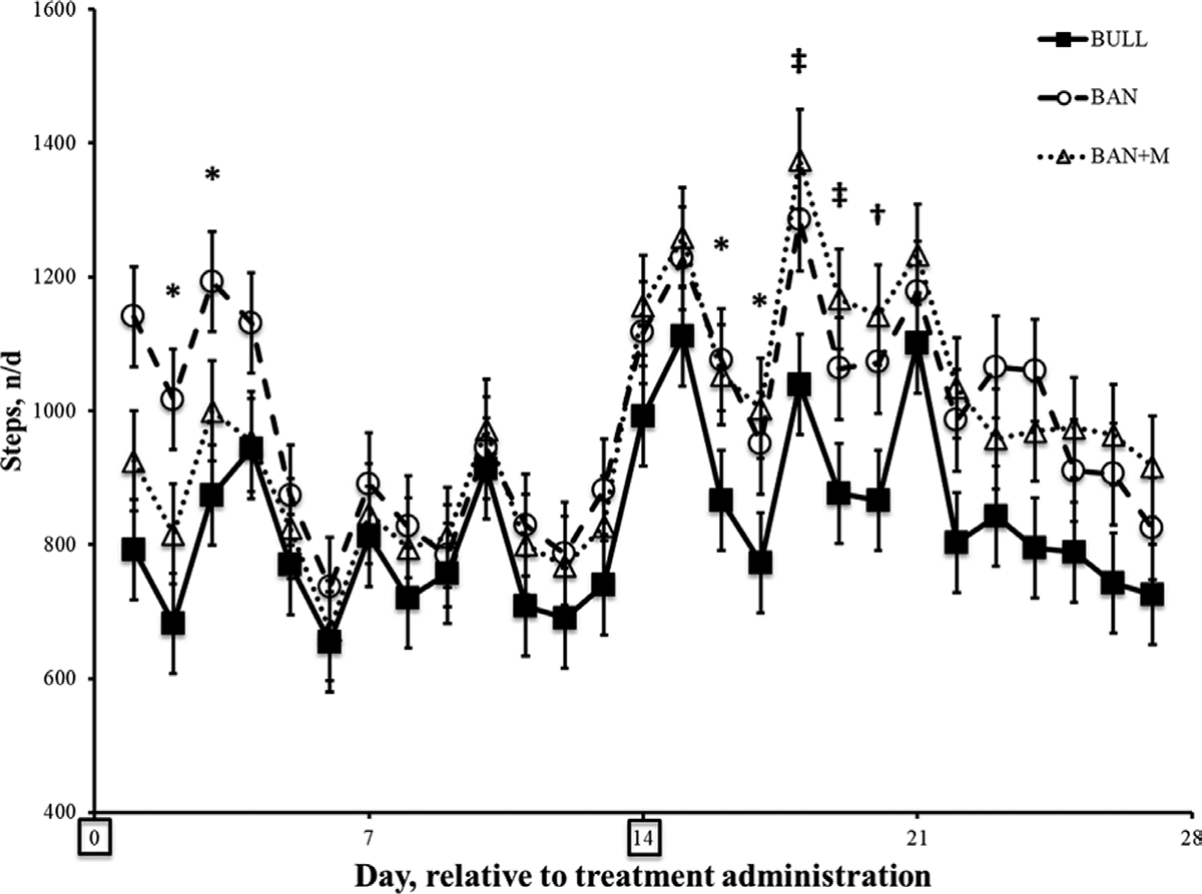
Steps (n/d ± SEM) recorded by IceTags (a three-axis accelerometer) on weaned weaned bulls (BULL; n = 16), bulls castrated on Day 0 using a Callicrate Bander (BAN; n = 16) and bulls castrated on Day 0 using a Callicrate Bander and administered meloxicam orally (3 mg/kg; BAN + M; n = 16) on Day 0 and 14 (boxed on the x-axis indicating the days of treatment). *Indicates BULL is less than BAN on the same day (P < 0.05). ‡Indicates BULL is less than BAN and BAN + M on the same day (P < 0.05). †Indicates BULL is less than BAN + M on the same day (P < 0.05).

Steps per day tended to differ among treatments (P = 0.09) with BULL taking fewer steps (829 ± 13 n/d) compared to BAN (991 ± 18 n/d) and BAN + M (972 ± 16 n/d). There was also an effect of day (P < 0.0001) and an interaction of treatment and day (P = 0.02). During the 3 and 6 d after the first (Day 0) and second (Day 14) administration of Mel, respectively, pairwise comparisons showed that on Days 2, 3, 16, and 17, BULL took fewer steps than BAN (P < 0.05), but did not differ significantly from BAN + M (P > 0.05; Figure 9).

**Figure 10.**
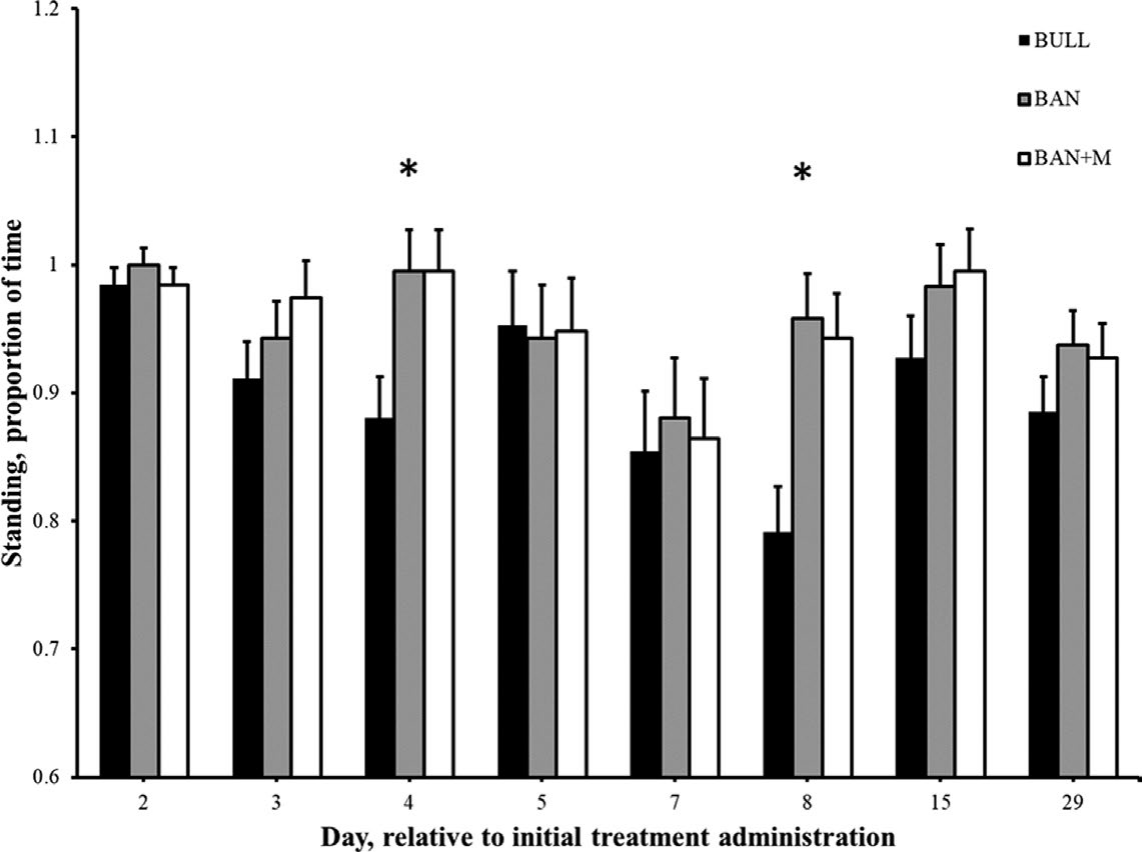
Proportion of time (+ SEM) standing (based on the ethogram) between 8 and 10 a.m. in weaned bulls (BULL), bulls castrated on Day 0 using a Callicrate Bander (BAN) and bulls castrated on Day 0 using a Callicrate Bander and administered meloxicam orally (3 mg/kg; BAN + M) on Day 0 and 14. *Indicates BULL is less than BAN and BAN + M on the same day (P < 0.05).

**Figure 11.**
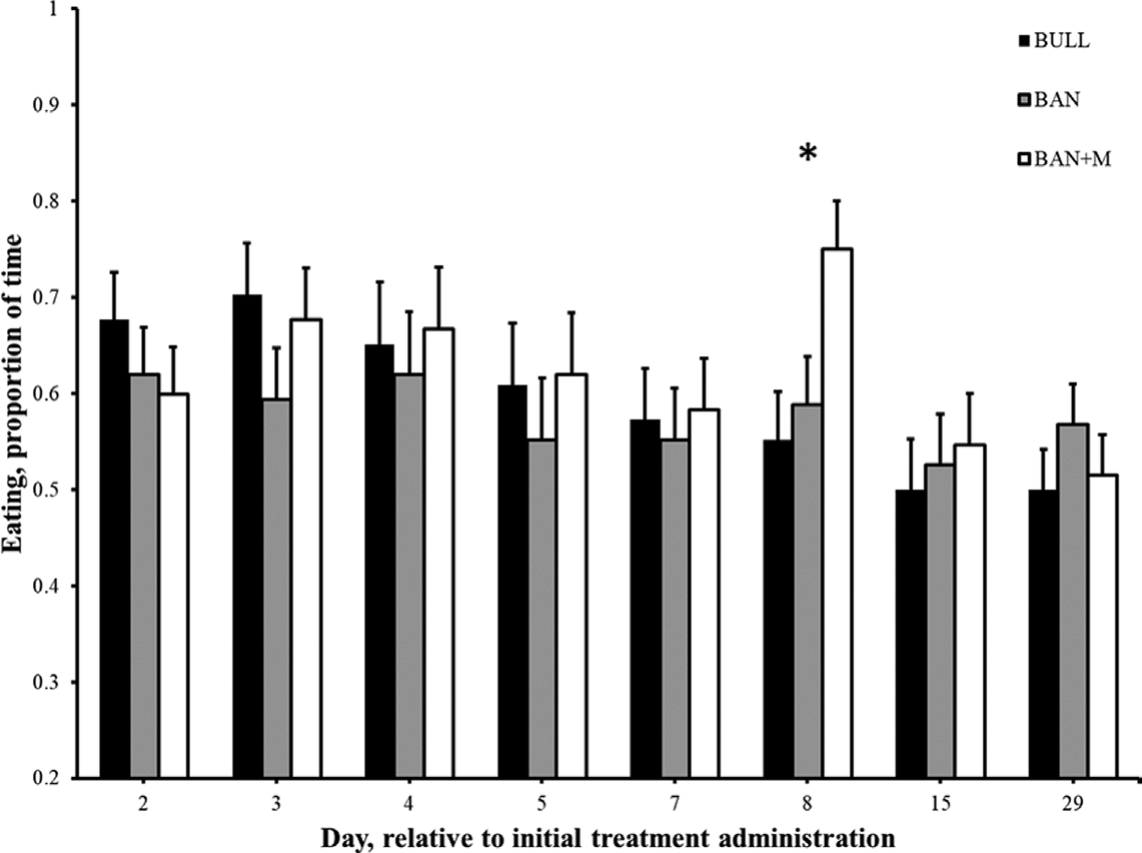
Proportion of time (+ SEM) eating (based on the ethogram) between 8 and 10 a.m. in weaned bulls (BULL), bulls castrated on Day 0 using a Callicrate Bander (BAN) and bulls castrated on Day 0 using a Callicrate Bander and administered meloxicam orally (3 mg/kg; BAN + M) on Day 0 and 14. *Indicates BAN + M is greater than BULL and BAN on the same day (P < 0.05).

There was an effect of treatment on percent of time calves spent standing (from 0800 to 0950 hour) on Day 4 (P = 0.006) and Day 8 (P = 0.0005), such that BULL spent a lesser percent of the time standing than BAN and BAN + M (Figure 10). To explore potential diurnal patterns in calf behavior, standing time was evaluated by splitting the morning observation period into two hourly categories: 0800 to 0850 hour and 0900 to 0950 hour. A significant effect of time category was observed, such that calves stood a greater percent of time during the first hour compared to the second hour on Day 5 (98.6 ± 0.83 and 91.0 ± 3.3%; P = 0.01), Day 7 (94.8 ± 1.6 and 78.5 ± 4.5%; P = 0.0009), Day 8 (95.1 ± 2.2 and 84.4 ± 3.4%; P = 0.004) and Day 29 (98.6 ± 1.4 and 84.7 ± 2.7%; P < 0.0001). There was no interaction between treatment and time category, indicating that these within-day behavioral patterns were not dependent on treatment group. This temporal analysis was exploratory in nature and intended to provide context for behavioral rhythms. There was an effect of time category, such that calves stood a greater percent of the time during the first hour (0800 to 0850 hour) as compared to the second hour (0900 to 0950) on However, there was no interaction (P ≥ 0.23) of treatment and time category (first or second hour) on percent of time calves spent standing. There was an effect of treatment on percent of time calves spent eating only on Day 8 (P = 0.002), such that BAN + M (75.0 ± 4.5%) spent a greater percent of time eating than BULL (55.2 ± 6.7%) and BAN [58.8 ± 4.4% (Figure 11)]. There was a significant effect of time category (first vs. second hour) on each day (P ≤ 0.002), with calves spending a greater percentage of time eating during the first hour (0800 to 0850 hour) compared to the second hour (0900 to 0950 hour). During the first hour, the mean percent time spent eating was as follows: Day 2 = 79.2 ± 3.2%, Day 3 = 77.4 ± 3.8%, Day 4 = 73.6 ± 3.7%, Day 5 = 66.7 ± 3.5%, Day 7 = 68.1 ± 3.2%, Day 8 = 73.3 ± 3.5%, Day 15 = 72.9 ± 3.2%, and Day 29 = 69.4 ± 3.4%. In contrast, during the second hour, the values were lower: Day 2 = 47.2 ± 3.8%, Day 3 = 54.2 ± 4.2%, Day 4 = 55.6 ± 4.0%, Day 5 = 51.0 ± 3.8%, Day 7 = 45.8 ± 4.4%, Day 8 = 52.8 ± 3.9%, Day 15 = 31.9 ± 3.5%, and Day 29 = 36.1 ± 3.4%. However, there was no interaction (P ≥ 0.36) of treatment and time category on percent of time calves spent eating. There was an effect treatment on the location within the pen (resting area, front of pen, or bunk; Figure 1) where calves spent their time only on Day 8 (P = 0.0004), such that BAN + M (75.5 ± 4.5%) spent a greater percent of time at the bunk than BULL (56.8 ± 7.0%) and BAN (59.4 ± 4.2%). There was an effect of time category on each day (P ≤ 0.03), such that calves spent a greater percent of their time in different locations within the pens during the first hour (0800 to 0850 hour) as compared to the second hour (0900 to 0950 hour). For example, during the first hour (0800 to 0850 hour), the mean percent time calves spent in the front of their pen was as follows: Day 2 = 20.1 ± 3.1%, Day 3 = 16.7 ± 3.3%, Day 4 = 21.2 ± 3.4%, Day 5 = 21.9 ± 2.9%, Day 7 = 18.4 ± 2.9%, Day 8 = 14.2 ± 2.8%, Day 15 = 18.4 ± 2.9%, and Day 29 = 22.9 ± 3.3%. In contrast, during the second hour, the values were greater (P < 0.05): Day 2 = 37.2 ± 3.4%, Day 3 = 27.4 ± 3.6%, Day 4 = 33.0 ± 3.8%, Day 5 = 38.2 ± 3.9%, Day 7 = 34.4 ± 4.5%, Day 8 = 28.1 ± 4.0%, Day 15 = 35.4 ± 4.1%, and Day 29 = 56.3 ± 3.4%. However, there was no interaction (P ≥ 0.40) of treatment and time category on where calves spent their time within the pens.

#### Rectal temperature.

**Figure 12.**
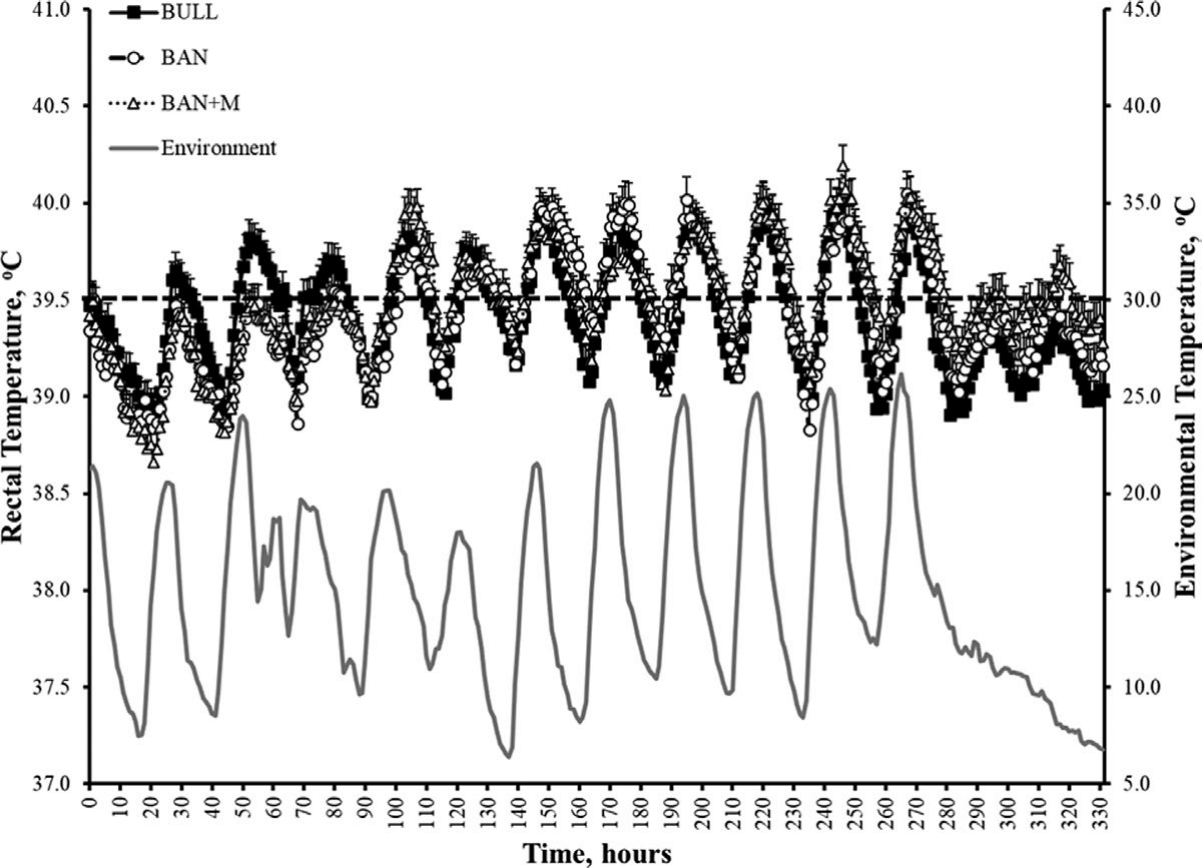
Environmental and rectal temperatures in a study of weaned bulls (BULL), bulls castrated on Day 0 using a Callicrate Bander (BAN) and bulls castrated on Day 0 using a Callicrate Bander and administered meloxicam orally (3 mg/kg; BAN + M) on Day 0 and 14. The temperature probes were left in place for approximately the first 14 d of the study. The dashed line (39.5 °C) is considered to be the minimum rectal temperature at which animals are febrile for the purpose of this study.

There was an effect of day (P < 0.0001) but no effect of treatment (P = 0.43) or interaction of treatment and day (P = 0.08) on rectal temperature during the 14 d after treatment administration (Figure 12). As part of an exploratory analysis examining behavioral influences on thermoregulation, we identified significant effects of standing (P < 0.0001) and lying time (P = 0.0009), as measured by IceTag™, on rectal temperature. Specifically, as standing time increased, rectal temperature decreased (β = –0.00245), whereas increased lying time was associated with a slight increase in rectal temperature (β = 0.000467).

While there was no effect of treatment (P = 0.59), there was an effect of day (P < 0.0001) and an interaction of treatment and day (P = 0.02) on the percent of time animals had a rectal temperature ≥ 39.5 °C (defined as febrile for the purpose of this study). BULL, BAN, and BAN + M were febrile 36.6 ± 5.3%, 42.7 ± 5.6%, and 43.0 ± 5.0% of the time, respectively. There were effects of standing time (P < 0.0001) and lying time (P = 0.03) on the probability that calves would be febrile, such that as standing and lying times increased, the percent of time animals had a fever decreased (−0.01899) and increased (0.004023), respectively.

## DISCUSSION

Band castration of weaned calves is a common procedure in beef production which is known to cause noxious stimuli and alter production related factors that can affect profitability and increase susceptibility to disease (Massey et al., 2011). Results from the current study show that Mel has an impact on behavior and band castration has an effect on rectal temperature in weaned calves post banding. Results also supported previously published data that band castration negatively impacts growth rate, influences the activity level of calves, and increases inflammatory markers like fibrinogen and haptoglobin (Pang et al., 2006; Repenning et al., 2013). In addition, this is one of the first reports that evaluated plasma concentration of Mel 2 wk post administration at a dose of 3 mg/kg.

Mel was administered orally at a dose of 3 mg/kg on Days 0 and 14. This dose was selected based on the prolonged effects associated with band castration in older calves (Fisher et al., 2001; Bretschneider, 2005) and the need for extended analgesic coverage beyond that typically achieved with a single dose (Coetzee et al., 2009). Previous studies evaluating a dose of 2 mg/kg Mel in calves showed mixed results (Daniel et al., 2020; Cull et al., 2022). One study in pre-weaned calves (90 to 100 d of age) undergoing band castration reported no differences in ADG or inflammatory markers, and only minor behavioral effects with Mel treatment (Daniel et al., 2020). Another study in weaned calves (~176 kg BW) undergoing surgical castration demonstrated transient improvements in behavioral pain scores and a modest increase in ADG over 14 d (Cull et al., 2022). However, the effects of Mel at this dose appear to be short-lived, particularly in procedures involving delayed tissue necrosis such as banding.

Pharmacokinetic data support the rationale for this dosing regimen. Coetzee et al. (2009) reported that a 1 mg/kg oral dose of Mel in calves (~150 to 170 kg BW) achieved a peak plasma concentration of ~3.1 μg/mL with a long half-life (~27 hours), and bioavailability near 100%. In comparison, studies using cumulative doses of 2 mg/kg (e.g., 1 mg/kg on Day −1 and two 0.5 mg/kg doses on Days 0 and 1) in band-castrated calves produced plasma concentrations of ~1.40 ug/mL on Day 0, which declined rapidly to ~0.001 ug/mL by Day 7 or 8, levels likely insufficient to provide analgesia throughout the healing period. In our study, the 3 mg/kg dose produced plasma Mel concentrations of 7.22 ± 0.57 μg/mL on Day 3 and 0.65 ± 0.15 μg/mL on Day 7, which are consistent with and exceed levels predicted by previous pharmacokinetic studies, given the higher dose administered (Repenning et al., 2013). These results suggest that the dosing regimen achieved the expected pharmacologic exposure and may have extended the window of effective analgesia beyond that previously reported for lower doses.

The 3 mg/kg dose was therefore selected to improve and extend analgesic efficacy during the early inflammatory phase, and a second dose at Day 14 was included to address potential discomfort during later stages of healing. This two-dose protocol was also selected for its practicality in commercial cattle operations, where repeated daily administration, as done in previous studies (Repenning et al., 2013), is often not feasible.

Resting and walking behaviors did not differ between BULL and BAN + Mel. This finding suggests that BAN + Mel calves may have experienced less pain to BAN calves post banding. This supports previous reports that the plasma concentrations of Mel > 0.2 µg/mL is considered adequate for analgesic effects as reported in horses with induced arthritis (Toutain and Cester, 2004). Though not directly translatable due to species variability and cause of noxious stimuli, bioavailability of oral Mel, as evident with plasma concentrations following a dose of 3 mg/kg BW (Figure 4), appeared to adequately achieve this previously determined therapeutic level in beef bulls for up to 7 d after treatment administration. Although Mel had a no impact on growth, indicators of systemic inflammation, or rectal temperature, Mel increased time laying down and number of steps taken while standing the few days following administration. This suggests that Mel may have decreased pain and inflammation in BAN + Mel calves. The reduction in painful behaviors by Mel in banded calves has been previously reported, even at lower dosing of 1 mg/kg (Olson et al., 2016). This supports that there may be future application of Mel administration prior to castration with positive implications for improving animal welfare.

It is important to note that behavioral observations in the present study were conducted during the morning period (0800 to 1000 hour), which coincided with the daily feeding time. This overlap may have influenced calf behavior, particularly oral activity, bunk attendance, and locomotion, due to anticipation or response to feed delivery. Although this time window was selected for consistency and to capture early-day behavior potentially reflective of discomfort or recovery, feeding-related activity could have confounded some treatment effects. Similar research evaluated the effects of band castration and Mel on behavior in weaned beef calves (Repenning et al., 2013). In that study, behavioral observations were performed in both the morning and afternoon, potentially providing a broader assessment of activity. However, the exact timing of feed delivery relative to behavioral observations was not specified, making it difficult to assess how feeding influenced the results. In future studies, conducting observations at multiple time points throughout the day, particularly outside of feeding periods, would provide a more comprehensive view of behavior and better isolate treatment effects from feeding-related activity.

Ethogram data for two hours (0800 to 0950 hour) on Day 4 and 8 post-castration revealed an impact of castration on percentage of time animals were standing. Oral Mel administration had negligible impact on pen-level behaviors documented with the ethogram at any point during the 2 hours observation period multiple days after castration. Results from the current study differ from those of a previous study that examined the effects of oral Mel (0.5 mg/kg) at dehorning in Holstein calves in which Mel-treated calves spent a greater percentage of time in recumbence than placebo control calves on Days 1, 2, 3, and 4 post dehorning (Theurer et al., 2012). A different study showed that preemptive Mel administration at 0.5 mg/kg in beef cows post cesarean section also increased lying behavior (Barrier et al., 2014). The inconsistency between the presented study and these two could be attributed to the acute pain associated with scoop dehorning and surgical pain versus band castration, which involves progressive necrosis of the scrotum and testis. A previous study using Angus bulls examined the effect of xylazine caudal epidural and flunixin meglumine in association with band castration and found no differences due to the effects of castration or NSAID medication on percent of cattle lying (Gonzalez et al., 2010). Similarly, another study found no effect of Mel on percentage of cattle lying or walking using a 0.5 and 1 mg/kg dosages (Repenning et al., 2013). This warrants further investigation into the analgesic dose of Mel needed in calves to reach therapeutic concentrations when investigating its use in reducing noxious stimuli related to castration.

The observed relationship between band castration and the percent of time calves were febrile may be influenced by changes in standing and lying behavior, although a direct causal relationship was not established in the present study. To the authors knowledge, the effects of standing and lying time on rectal temperature in relation to band castration have not been previously reported. Heat stress is known to compromise immune responses in calves which can lead to onset of respiratory and gastrointestinal disease, in turn triggering decreased ADG and increased profit loss (Wang et al., 2020; Yu et al., 2024). When calves are standing, they have increased heat dissipation secondary to increased body surface area exposure for thermal convection and radiation, compared to when they are laying down and experience more conduction with the ground and subsequently are unable to dissipate heat as easily. Understanding the inverse relationship of standing time and rectal temperature in relation to band castration in postweaning calves could help with future management practices to reduce stress, secondary medical conditions, and profit loss.

Castration had an adverse impact on ADG in beef bulls, with BULL exhibiting greater ADG than castrates, similar to previous studies where castrates exhibited reduced ADG when compared to intact bulls (Fisher et al., 2001). Results from this study revealed that oral Mel had no impact on ADG in weaned calves following band castration, similar to results reported in pre-weaned calves (Daniel et al., 2020). These results also concur with findings of a previous study examining oral Mel around the time of band castration where Mel had no impact on ADG (Repenning et al., 2013).

This study dosage frequency was one dose every 2 wk for two administrations. This can be seen as a limitation of the study in practicality because it may not be realistic that producers are able to catch the animals again for a second oral dose administration, depending on availability of personnel and facilities. Additionally, rectal probes remained in place for a 2-wk period. It is logical that the probes may have stimulated a level of iatrogenic inflammation of the rectum which could have contributed to increased temperatures and inflammation associated biomarkers reported. However, the rectal probe placements were performed by trained personnel and rectums and tails were observed for trauma daily (Reuter et al., 2010).

Though Mel administered at 3mg/kg orally 2 wk apart did not have a significant impact on ADG or inflammatory markers in castrates, the increased lying time and steps while standing suggest that Mel may have decreased certain pain behaviors to that of an uncastrated bull up to 3 d post banding with initial Mel dose, and up to 6 d following the second Mel dose. Future studies of Mel should investigate the appropriate analgesic dose and frequency necessary to reduce pain associated biomarkers, improve ADG for band castration in postweaning calves, or explore other analgesic options.
